# Utilizing Optimized Tools to Investigate PTM Crosstalk: Identifying Potential PTM Crosstalk of Acetylated Mitochondrial Proteins

**DOI:** 10.3390/proteomes6020024

**Published:** 2018-05-22

**Authors:** Henrick Horita, Andy Law, Kim Middleton

**Affiliations:** Research and Development Department, Cytoskeleton Inc., Denver, CO 80223, USA; andyl@cytoskeleton.com (A.L.); kimm@cytoskeleton.com (K.M.)

**Keywords:** post-translational modification, acetylation, ubiquitination, SUMOylation, PTM crosstalk, mitochondria acetylation, PDHA1, IDI1, ATIC, PDHB

## Abstract

Post-translational modification (PTM) crosstalk is recognized as a major cell-regulatory mechanism, and studies of several proteins have validated the premise that PTMs work in concert. Previous work by our group investigated the potential PTM crosstalk on proteins in the EGFR-Ras-c-Fos axis by utilizing a comprehensive set of PTM reagents termed Signal-Seeker toolkits. In this study, these tools were used to investigate the potential PTM crosstalk that occurs in acetylated mitochondrial proteins in response to a mitochondrial stress-inducing agent hydrogen peroxide (H_2_O_2_). Mitochondrial protein acetylation has been shown to participate in PTM crosstalk as exemplified by the regulation of the pyruvate dehydrogenase complex via kinase, phosphatase, acetyltransferase, and deacetylase activities. Changes in the acetylated state of mitochondrial proteins were investigated, in response to H_2_O_2_, using a novel anti acetyl lysine (Ac-K) antibody. Signal-Seeker PTM detection tools were used to validate the acetylation state of ten mitochondrial targets, as well as their endogenous acetylation state in response to H_2_O_2_. Importantly, the endogenous acetylation, ubiquitination, SUMOylation 2/3, and tyrosine phosphorylation state of four target mitochondrial proteins were also investigated with the toolkit. Each of the four proteins had unique PTM profiles, but diverging acetylation and ubiquitin or SUMO 2/3 signals appeared to be a common theme. This proof-of-concept study identifies the Signal-Seeker toolkits as a useful tool to investigate potential PTM crosstalk.

## 1. Introduction

Post-translational modifications (PTMs) are dynamic, tightly-regulated alterations to a protein that control the protein’s structure, spatial localization, and interactions; thereby, governing its function [1,2,3]. Well-studied PTMs include, acetylation (Ac), ubiquitination (Ub), small ubiquitin-like modifier 2/3 (SUMOylation 2/3 (SUMO 2/3)), and tyrosine phosphorylation (pY), but there are many others [4,5,6,7,8]. Unbiased approaches such as mass spectrometry (MS)-proteomics have championed the growth of the PTM field by unveiling the vast abundance of PTM targets [9]. Individual PTMs, and importantly, combinations of PTMs (i.e., PTM crosstalk) increase the number of unique proteoforms from roughly 30,000 to potentially millions [10,11]. PTM crosstalk is recognized as a major cell regulatory mechanism [12,13], and extensive molecular studies of well-characterized proteins like p53, tau, and tubulin have validated the premise that PTMs work in concert to regulate these protein’s functions [14,15,16]. The idea of PTMs working together to alter the protein’s regulation and function was popularized by the epigenetic field, and termed the histone code [17]. Recent PTM studies of other protein targets further support the hypothesis of both positive and negative PTM crosstalk as regulatory mechanisms [18,19,20,21]. As investigators continue to decipher the role that PTMs play in regulating their target protein of interest, it will be paramount to characterize the temporal regulation and interplay of PTMs and their crosstalk.

While it is becoming clear that PTM crosstalk constitutes a major regulatory mechanism for protein function, mass spectrometry approaches [22,23,24,25], employed to identify potentially important quantitative PTM changes on any given protein/pathway, are highly specialized and not yet readily available to most labs. We have previously reported a simple approach that allows non-PTM specialists to take a snapshot of potential roles of PTMs in their system [26,27,28]. The Signal-Seeker toolset utilizes powerful affinity reagents for a range of common PTMs including Ac, pY, SUMO 2/3 and Ub, along with a universal lysis buffer that allows simultaneous analysis of these PTMs from a single lysate [28]. PTMs are detected using Western blot analysis, offering a quick, sensitive and easy way to determine if PTMs or PTM crosstalk could play a role in the regulation of any protein/system of interest.

This report applies the Signal-Seeker technology to study potential crosstalk between acetylation and other PTMs in mitochondria. Mitochondrial protein acetylation has been shown to participate in PTM crosstalk as exemplified by the regulation of the pyruvate dehydrogenase complex via kinase, phosphatase, acetyltransferase, and deacetylase activities [29]. Changes in the acetylated state of mitochondrial proteins were investigated, in response to hydrogen peroxide (H_2_O_2_) stimulation, using a novel anti acetyl lysine (Ac-K) antibody. Signal-Seeker PTM detection tools were used to detect acetylation of the mitochondrial protein, PDHA1, as well as changes to its endogenous acetylation state in response to H_2_O_2_, which tracked with mitochondrial acetylation changes observed by IF. These PTM detection tools were also used to examine PDHA1 Ub, pY, and SUMO 2/3, and an increase in PDHA1 Ub was observed concomitantly with the decreased acetylation in response to H_2_O_2._ Preliminary proteomic studies identified an array of acetylated mitochondrial protein targets immunoprecipitated with the Ac-K antibody, and validation of 10 key targets were performed with Signal-Seeker PTM detection methodologies. Changes in acetylation in response to H_2_O_2_ for the 10 targets were also examined. Finally, crosstalk of Ac, Ub, SUMO 2/3, and pY modifications were analyzed for 4 of these mitochondrial targets. This proof-of-concept study highlights the simplicity and power of the Signal-Seeker toolkit to allow non-PTM/proteomics specialists to gain great insight into how/if PTMs may be affecting their protein/system of interest.

## 2. Materials and Methods

### 2.1. Cell Culture and Reagents

A431 human epidermoid carcinoma cells and 3T3-swiss mouse fibroblast cells were obtained from ATCC. Cells were grown in DMEM media (ATCC, Manassas, VA, USA) supplemented with 10% FBS (Atlas Biologicals, Fort Collins, CO, USA) and penicillin/streptomycin (ThermoFisher, Waltham, MA, USA). TrypLE Express was obtained from Gibco (ThermoFisher, Waltham, MA, USA). Unless otherwise noted, chemicals were obtained from Sigma Chemical Co. (Sigma, St. Louis, MO, USA). For all Western blot and IP analysis experiments, A431 cells at 60% confluency, post 5 days of cell passage, were treated with 100 μM H_2_O_2_ for 2 h in individual 15 cm dishes (Corning, Corning, NY, USA).

### 2.2. Immunization of Mice

AAC03 was generated using a traditional immunization method. Briefly, six-week-old BALB/c mice (Envigo, Indianapolis, IN, USA) were injected intraperitoneally with 50 μg of a proprietary mixture of acetylated proteins emulsified in complete Freund’s adjuvant (ThermoFisher, Waltham, MA, USA). Three subsequent injections were performed at 3 weeks intervals with the same mixture of acetylated proteins in incomplete Freund’s adjuvant. Splenocytes were collected and fused with P3X63Ag8.653 mouse myeloma cells (ATCC, Manassas, VA, USA) using polyethylene glycol 1500 (PEG 1500, ATCC, Manassas, VA, USA). Hybridoma supernatants were screened by ELISA, and positive wells were subcloned by limiting dilution and single-cell selection. AAC02 was generated using a repetitive immunization method. Six-week-old BALB/c mice (Envigo, Indianapolis, IN, USA) were injected subcutaneously with 20 μg of a proprietary mixture of acetylated proteins emulsified in complete Freund’s adjuvant and RIBI (Sigma, St. Louis, MO, USA). Five subsequent injections were performed with the same mixture of acetylated proteins in incomplete Freund’s adjuvant and RIBI. Antibody producing cells were collected and fused with P3X63Ag8.653 mouse myeloma cells using PEG 1500. Hybridoma supernatants were screened by ELISA, and positive wells were subcloned by limiting dilution and single-cell selection.

### 2.3. Western Blotting

A431 cells, treated or untreated with 100 μM H_2_O_2_, were lysed with ice-cold BlastR lysis buffer containing a cocktail of Trichostatin A (TSA) (1 μM), nicotinamide (16.5 mM), and protease inhibitors (PIC02), or n-ethylmaelimide (NEM) or Sodium Orthovanadate (Na_3_VO_4_). BlastR lysis buffer is a complete cell lysis reagent that is comprised of a proprietary mixture of detergents, salts, and other buffer additives. DNA was removed by passing the lysate through the compressible BlastR filter system (Cytoskeleton, Denver, CO, USA). After dilution with BlastR dilution buffer, protein concentrations were determined with Precision Red Advanced protein reagent (Cytoskeleton, Denver, CO, USA), and measured at 600 nm OD. Protein lysate samples were separated using Tris-glycine SDS-polyacrylamide gel electrophoresis (ThermoFisher, Waltham, MA, USA) and transferred to Immobilon- P membranes (Millipore, Burlington, MA, USA). Membranes were blocked for 30 min at room temperature in Tris-buffered saline (10 mM Tris-HCl, pH 8.0, 150 mM NaCl) containing 0.05% Tween-20 (TTBS) and 5% milk (Thrive Life, American Fork, UT, USA), and then incubated with 0–2.5% milk in TTBS solution containing primary antibodies for 1–2 h at room temperature (RT). Membranes were washed in TTBS 3 × 10 min, prior to secondary antibody for 1 h at RT. Bound antibodies were visualized with horseradish peroxidase-coupled secondary antibodies and chemiluminescent reagent (Cytoskeleton, Denver, CO, USA) according to the manufacturer’s directions. Antibodies used: PDHA1 (18068-1-AP; 1:1000, Proteintech, Rosemont, IL, USA), PDHB (14744-1-AP; 1:1000, Proteintech, IL), PRDX2 (10545-2-AP; 1:1000, Proteintech, IL), IDI1 (11166-2-AP; 1:1000, Proteintech, IL), MRPL24 (16224-1-AP; 1:1000, Proteintech, IL), DTYMK (15360-1-AP; 1:500, Proteintech, IL), Hexokinase 2 (22029-1-AP; 1:1000, Proteintech, IL), SSBP1 (12212-1-AP; 1:500, Proteintech, IL), DLD (16431-1-AP; 1:500, Proteintech, IL), ALDH9A1 (26621-1-AP; 1:1000, Proteintech, IL), ATIC (A304-271A; 1:1000, Bethyl Laboratories, Montgomery, TX, USA), and HRP-anti-rabbit secondary (1:10,000; Jackson ImmunoResearch, West Grove, PA, USA). Changes were quantitated by densitometry using Image J software (rsb.info.nih.gov).

### 2.4. Immunoprecipitation (IP) Assay

A431 cells, treated or untreated with 100 μM H_2_O_2_, were lysed with ice-cold BlastR lysis buffer containing a cocktail of TSA (1 μM), nicotinamide (16.5 mM), and PIC02, or NEM or Na_3_VO_4_. DNA was removed by passing the lysate through the BlastR filter system (Cytoskeleton, Denver, CO, USA). After dilution with BlastR dilution buffer, protein concentrations were determined with Precision Red Advanced protein reagent (Cytoskeleton, Denver, CO, USA), and measured at 60 m OD. Samples were immunoprecipitated, using Signal-Seeker kits, with equal protein concentration and IP volumes according to the manufacturer’s protocol (Cytoskeleton, Denver, CO, USA). The recommended amount of pY (30 μL/g lysate; APY03-beads), Ub (20 μL/g lysate; UBA01-beads), SUMO 2/3 (40 μL/g lysate; ASM24-beads), Ac (50 μL/g lysate; AAC04-beads), Acetyl-lysine IgG control beads (50 μL/g lysate; CIG02-beads), IgG control beads (30–40 μL/g lysate; CIG01-beads), or Ub control beads (20 μL/g lysate; CUB01-beads) were added to the respective samples for 1–2 h and rotated at 4 °C. After incubation, the affinity beads from each sample were pelleted and washed 3X with BlastR wash buffer. Bound proteins were eluted using bead elution buffer (Cytoskeleton, Denver, CO, USA) and processed by Western immunoblotting.

### 2.5. Immunofluorescence (IF) Assay

Swiss 3T3 cells were seeded on glass coverslips for at least 24 h before treatment. Swiss 3T3 cells were either untreated or treated with 1 μM of TSA for 6 h. Competition experiments were performed by adding 10 μg/mL of acetylated BSA to the AAC02 and AAC03 antibody incubation step. Organelle co-localization experiments were performed by addition of 10 M Mitotracker orange (Thermofisher, Waltham, MA, USA) to cells for 30 min prior to fixation, or co-staining with LAMP-1 antibody (Abcam, Cambridge, MA, USA). Dynamic mitochondrial acetylation experiments were performed by treating A431 cells with 100 μM of H_2_O_2_ for 2, 6, and 24 h.

After respective treatments, cells were washed with 1xPBS and fixed with 4% formaldehyde for 10 min followed by permeabilization with 0.5% Triton X-100 for 15 min. Cells were then blocked with 2% BSA/PBST/5% normal goat serum for 30 min. Cells were incubated with acetylation antibodies (AAC02; 1:1000, Cytoskeleton Inc., Denver, CO, USA) or (AAC03; 1:1000, Cytoskeleton Inc., Denver, CO, USA), or lysosomal marker LAMP 1 (ab24170; 1:100, Abcam, Cambridge, MA, USA) that were diluted with 2% BSA/PBST and incubated on fixed cells for 1 h. Acetylation signals and lysosomal signals were visualized with goat anti-mouse Alexa 488 (1:1000) and goat anti-rabbit Alexa 555 (1:1000) respectively (Thermofisher, Waltham, MA, USA). Cells were incubated with DAPI stain for 5 min and washed three times with 1xPBS. Coverslips were mounted on slides (Thermofisher, Waltham, MA, USA) using Fluro-Gel mounting media (Electron Microscopy Sciences, Hatfield, PA, USA).

Images were acquired with a Nikon 100×/1.30 Plan Fluor objective lens on a Nikon Eclipse E600 microscope. For each antibody labeling condition, the image acquisition settings were kept the same between different experiments. The brightness, contrast, and levels of the images were adjusted and compiled using ImageJ (NIH) software (v. 1.41, National Institutes of Health, Bethesda, MD, USA, https://imagej.nih.gov/ij/index.html). No additional digital image processing was performed.

## 3. Results

### 3.1. Immunofluorescence Detection of Dynamic Changes in Mitochondrial Acetylation in Response to H_2_O_2_

**Figure 1:**
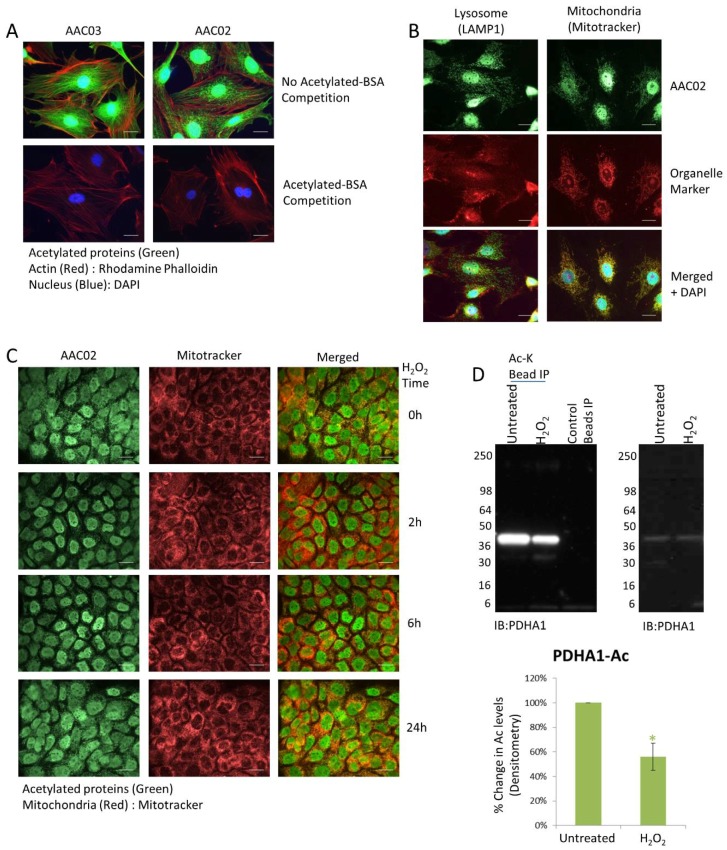
Ac-K Antibody, AAC02, detects dynamic changes in acetylated mitochondrial protein by IF. (**A**) Representative examples of TSA treated Swiss 3T3 cells stained with the Ac-K antibodies AAC02 or AAC03. Competition experiments (**bottom panels**) were performed by adding 10 μg/mL of acetylated BSA to the AAC02 and AAC03 antibody incubation step. Bars = 10 μm (**B**) Representative examples of untreated Swiss 3T3 cells co-labeled with AAC02 and a mitochondria marker (mitotracker) or AAC02 and a lysosomal marker (LAMP-1). Bars = 10 μm (**C**) Representative examples of A431 cells co-labeled AAC02 and mitotracker. The mitochondrial acetylation state of A431 cells was measured after treatment with 100 μM of H_2_O_2_ for 0, 2, 6, and 24 h. Bars = 10 μm (**D**) A431 cells either untreated or treated with H_2_O_2_ for 2 h were lysed with BlastR lysis buffer. IP of acetylated proteins from 800 μg of lysate were performed using Ac-K Affinity beads or Acetyl-lysine IgG control beads. Western blots, on eluted proteins, were performed with a PDHA1 antibody. Shown are representative Acetylation IP (**left**) and whole cell lysate (WCL) (**right**) Westerns from N ≥ 3 independent experiments. Quantification of background subtracted densitometric analysis of PDHA1 acetylation is shown. Error bars represent s.e.m. *t*-test statistical analysis was performed. * *p* < 0.05.

With the recent growth in Ac targets that span well beyond epigenetic regulation [30], there has been an increased demand for additional Ac research tools. To characterize and identify unique Ac targets and/or cellular functions, two pan-acetyl-lysine monoclonal antibodies (AAC02 and AAC03) were developed by immunizing mice with a mixture of acetylated proteins. While validating these tools for IF applications it was noted that AAC03 identified histones and acetylated tubulin in response to TSA treatment (Figure 1A), which is similar to other pan-acetyl-lysine antibodies. Surprisingly, AAC02 had a unique IF profile that appeared to identify proteins in vesicular or mitochondrial structures (Figure 1A). Proteins in the mitochondria, in particular, are highly acetylated [31]; thus, it was of interest to determine if AAC02 could track the Ac state of mitochondrial proteins by IF, which was not possible with the current group of commercial pan-acetyl lysine antibodies. IF assays were performed on 3T3 cells that were co-stained with AAC02 and a mitochondria marker, mitotracker; alternatively, 3T3 cells were co-stained with AAC02 and the vesicle marker, LAMP1 (Figure 1B). The data in Figure 1B shows convincing co-localization of AAC02 and mitochondrial structures, but minimal overlap with vesicles. While this data suggests that AAC02 identifies acetylated mitochondria proteins, it does not determine whether AAC02 can detect dynamic and physiologic changes of acetylated proteins in the mitochondria. To address this question, A431 cells were treated with H_2_O_2_, an oxidative agent that has been shown to regulate the mitochondrial de-acetylase, sirtuin 3 (SIRT3) [32]. Data in Figure 1C shows a pronounced decrease in mitochondrial acetylation staining with minimal changes in mitotracker staining at 2-h of treatment with H_2_O_2_, as well as subsequent recovery of the acetylation signal at later time-points. Collectively, these data suggest that AAC02 may be a useful tool for tracking changes in acetylated mitochondria by IF.

### 3.2. PDHA1 Exhibits Diverging Changes in Acetylation and Ubiquitination in Response to H_2_O_2_

To identify potential acetylated mitochondrial proteins recognized by AAC02, literature searches were performed and a promising target, pyruvate dehydrogenase E1 alpha 1 subunit (PDHA1), was identified [33]. PDHA1 was chosen because it is an acetylated mitochondrial protein that is dynamically regulated by de-acetylases [33]. Endogenous PDHA1 Ac was investigated using the Signal-Seeker PTM detection tool. Results in Figure 1D show that PDHA1 is highly acetylated under basal conditions, and in response to 2 h of H_2_O_2_ treatment, its acetylation state is reduced by 44%. These findings correlate with the decrease in mitochondrial acetylation observed in Figure 1C at 2 h and implicate PDHA1 as an acetylated mitochondrial target that is recognized by AAC02.

**Figure 2:**
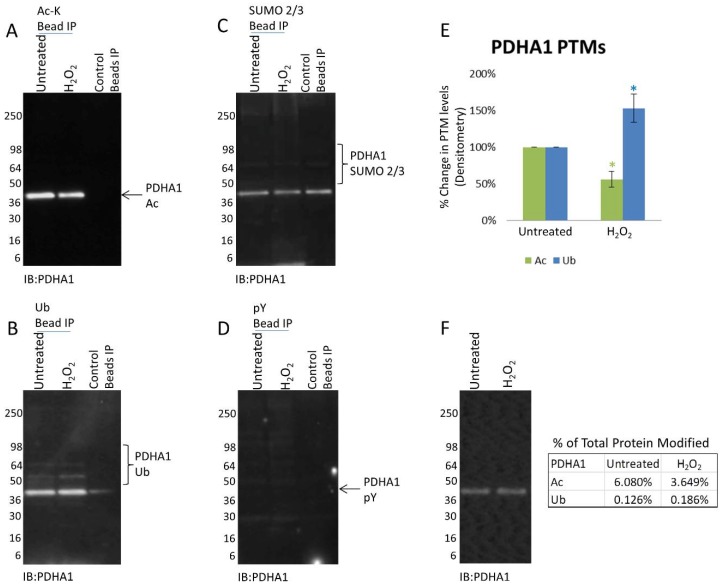
H_2_O_2_ induced Ac, Ub, SUMO 2/3, and pY modifications of PDHA1. A431 cells either untreated or treated with H_2_O_2_ for 2 h were lysed with BlastR lysis buffer. Untreated and treated A431 lysates were incubated with (**A**) Ac-K beads to IP acetylated proteins and analyzed for acetylated PDHA1, (**B**) Ub beads to IP ubiquitinated proteins and analyzed for ubiquitinated PDHA1, (**C**) SUMO 2/3 beads to IP SUMOylated 2/3 proteins and analyzed for SUMO 2/3 modified PDHA1, (**D**) and pY beads to IP tyrosine phosphorylated proteins and analyzed for tyrosine phosphorylated PDHA1. All IPs were performed with appropriate control beads to detect non-specific detection. Shown are representative Westerns from N ≥ 3 independent experiments. (**E**) Quantification of background subtracted densitometric analysis of PDHA1 PTMs. Error bars represent s.e.m. *t*-test statistical analysis was performed. * *p* < 0.05. (**F**) WCL was analyzed for PDHA1 levels. The percentage of PTM modified PDHA1 relative to the total PDHA1 levels for each modification is shown.

H_2_O_2_ is a broad cell stress inducing agent that can regulate cell processes such as kinase signaling [34] and does not exclusively regulate Ac; thus, it was possible that it may be activating additional PTMs on PDHA1. The Signal-Seeker tool allows investigators to look beyond single PTMs and examine potential PTM crosstalk of a target protein [28]; therefore it was used to determine if alternative PTMs were also activated on PDHA1 in response to H_2_O_2_. Endogenous Ub, SUMO 2/3 and pY modifications of PDHA1 were investigated in response to H_2_O_2_ in parallel with Ac (Figure 2). The data shows that H_2_O_2_ does not affect the SUMO 2/3 or pY state of PDHA1 (Figure 2C,D), however PDHA1 Ub increased by 53% after 2 h of treatment (Figure 2B). It is important to note that the percentage of total PDHA1 that is acetylated (6.08% and 3.649%) is significantly higher than the percentage of ubiquitinated PDHA1 (0.126% and 0.186%) (Figure 2F). Nevertheless, the data indicates that multiple PTMs of PDHA1 are simultaneously changing in response to H_2_O_2_ and provides an avenue of further investigation to decipher if these modifications of PDHA1 regulate each other.

### 3.3. H_2_O_2_ Treatment Results in the Deacetylation of Multiple Mitochondrial Proteins

To further validate the utility of the Signal-Seeker toolkits in identifying acetylated proteins in this system we used mass spectrometry (MS) to identify proteins that were immunoprecipitated using Signal-Seeker AAC02 acetyl lysine affinity beads. In this case, MS was not performed to identify specific acetylation sites as we did not IP acetylated peptides, rather whole proteins were immunoprecipitated followed by trypsin digestion for MS analysis. This low sensitivity strategy was employed to allow us to maximize the chances of identifying the Signal-Seeker protein targets as multiple peptides would be generated per target protein. These preliminary studies identified 1196 protein targets and over 200 mitochondrial proteins that were predicted to be acetylated (Supplementary File S1). Additional replicates are necessary before complete analysis and reporting on these findings are possible; however, ten mitochondrial targets were randomly chosen for further validation (Table 1). 

**Figure 3:**
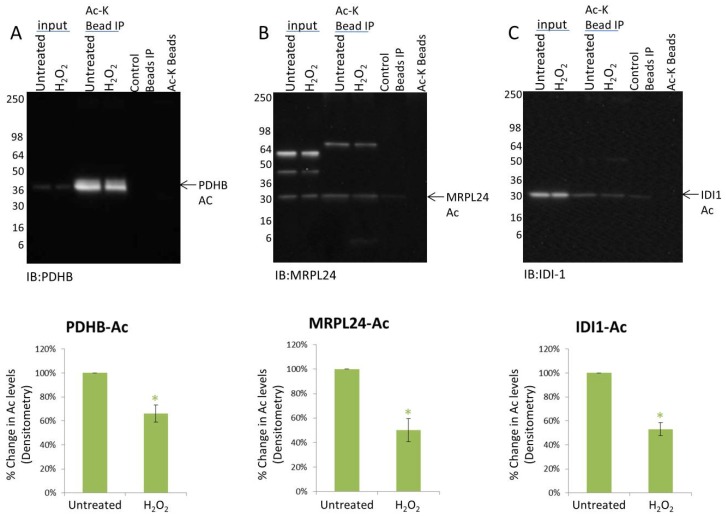
Endogenous acetylation of PDHB, MRPL24, and IDI1. A431 cells either untreated or treated with H_2_O_2_ for 2 h were lysed with BlastR lysis buffer. IP of acetylated proteins from 800 μg^−1^ mg of lysate were performed using Ac-K Affinity beads or Acetyl-lysine IgG control beads. Eluted proteins were resolved in an SDS-PAGE gel and then transferred to a PVDF membrane. Western blots were performed with (**A**) PDHB, (**B**) MRPL24, and (**C**) IDI1 antibodies. Shown are representative Westerns from N ≥ 3 independent experiments. Quantification of background subtracted densitometric analysis of the three proteins is shown. Error bars represent s.e.m. *t*-test statistical analysis was performed. * *p* < 0.05.

The ten protein targets were all predicted to have mitochondria localization according to the mitocarta 2.0 database [35] and 8 have been validated as mitochondrial localized [36,37,38,39,40,41,42,43] (Table 1). The majority of these proteins had been previously identified as acetylated by mass spectrometry [44]. Only PDHA1 and peroxiredoxin 2 (PRDX2) have previously been validated as acetylated by conventional methods [33,45]. The Signal-Seeker acetyl-lysine enrichment tool was used in conjunction with Western blot analysis to further validate that the ten mitochondrial targets were endogenously acetylated. As predicted, all 10 proteins were confirmed to be acetylated (Figure 3 and Figure S1). Additionally, the data presented here identified acetylation of mitochondrial ribosomal protein L24 (MRPL24) and hexokinase 2 (HK2) in human samples, which had not been detected previously (Figure 3B and Figure S1). Importantly, endogenous changes in the acetylated state of these 10 proteins in response to H_2_O_2_ were also investigated to determine if the Western data for these targets correlate with the IF results and to identify subjects for potential PTM crosstalk in response to H_2_O_2_. Data in Figure 3 shows pyruvate dehydrogenase E1 beta subunit (PDHB), MRPL24, and isopentenyl-diphosphate delta isomerase 1 (IDI1) acetylation state decreases in response to H_2_O_2_ by 33.9%, 49.9% and 47% respectively. H_2_O_2_-induced changes for all target proteins are shown in Table 1. Importantly, the data shows that a majority of the targets (70%) had significantly decreased acetylation states in response to H_2_O_2_. Collectively, these findings support the premise that AAC02 effectively detects changes in mitochondrial protein acetylation in both IF and IP applications, and that semi-quantitative changes in the PTM status of endogenous protein targets can be observed using Signal-Seeker tools and standard Western blot techniques.

### 3.4. Mitochondrial Proteins Exhibit Protein Specific PTM Changes in Response to H_2_O_2_

**Figure 4:**
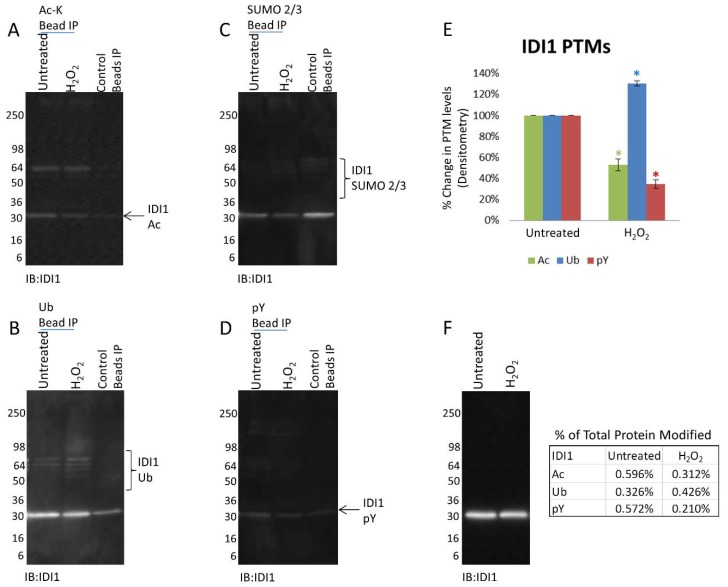
H_2_O_2_ induced Ac, Ub, SUMO 2/3, and pY modifications of IDI1. A431 cells either untreated or treated with H_2_O_2_ for 2 h were lysed with BlastR lysis buffer. Untreated and treated A431 lysates were incubated with (**A**) Ac-K beads to IP acetylated proteins and analyzed for acetylated IDI1, (**B**) Ub beads to IP ubiquitinated proteins and analyzed for ubiquitinated IDI1, (**C**) SUMO 2/3 beads to IP SUMOylated 2/3 proteins and analyzed for SUMO 2/3 modified IDI1, (**D**) and pY beads to IP tyrosine phosphorylated proteins and analyzed for tyrosine phosphorylated IDI1. All IPs were performed with appropriate control beads to detect non-specific detection. Shown are representative Westerns from N ≥ 3 independent experiments. (**E**) Quantification of background subtracted densitometric analysis of IDI1 PTMs. Error bars represent s.e.m. *t*-test statistical analysis was performed. * *p* < 0.05. (**F**) WCL was analyzed for IDI1 levels. The percentage of PTM modified IdI1 relative to the total IDI1 levels for each modification is shown.

Several of the mitochondrial proteins that exhibited decreased acetylation in response to H_2_O_2_ were further examined to assess their potential for PTM crosstalk. Utilizing the Signal-Seeker toolkit, pY, Ub, and SUMO 2/3 modifications were investigated and a summary of the results are reported in Table 2. Data in Figure 4 provides a snapshot of the PTM profile of IDI1 in response to H_2_O_2_ treatment. Analogous to PDHA1, IDI1 Ub increased (30.3%) in response to H_2_O_2_ while its Ac state decreased (47%) over the 2 h treatment period (Figure 4A,B). However, while pY of PDHA1 was not detected, pY of IDI1 was present in untreated cells, and significantly decreased (65.1%) in response to H_2_O_2_ (Figure 4D). In the case of IDI1, its Ac (0.596% and 0.312%), Ub (0.326% and 0.426%), and pY (0.572% and 0.210%) state appear to represent a similar percentage of the total IDI1 protein levels. To our knowledge, this is the first endogenous validation of these PTMs and, importantly, the first report showing that the IDI1 PTM levels change in a potentially coordinated manner in response to a given treatment. Another target, PDHB showed a distinct increase in SUMO 2/3 modification, and a trend towards increased Ub in response to H_2_O_2_ (Figure S2). The final target, 5-aminoimidazole-4-carboxamide ribonucleotide formyltransferase /IMP cyclohydrolase (ATIC), displayed an increase in SUMO 2/3 while no Ub was detected for the protein (Figure S3). These findings highlight the prevalence of PTMs on mitochondrial target proteins, and their potential for crosstalk.

## 4. Discussion

Common PTM crosstalk paradigms are beginning to emerge in the PTM field. For example phosphorylation has been shown to be a precursor to target protein Ub in several proteins [46,47]. Another pattern of PTM crosstalk exists between Ac and Ub where these modifications have been reported to compete to regulate the stability of the target protein [48,49]. Ac has been shown to stabilize target proteins [50], while poly-Ub is well-characterized as a mark for proteasomal degradation [5]. Both Ac and Ub primarily modify lysine residues, and many proteins have been identified that can be both acetylated and ubiquitinated on the same lysine residue [44], supporting the possibility of negative crosstalk between these PTMs.

Four mitochondrial proteins, PDHA1, IDI1, ATIC and PDHB were examined for potential crosstalk in this report. The activity of PDHA1 in regulating glucose homeostasis has previously been shown to be tightly regulated by PTM crosstalk, in particular between lysine acetylation (K321) and serine phosphorylation (S293) [29]. The data presented in this report supports a physiological role for PDHA1 acetylation as its endogenous Ac levels change in response to treatment with H_2_O_2_ (Figure 1D). Proteomic analysis has previously identified a single Ub modification (K385) on PDHA1 [51], we report an increase in ubiquitinated PDHA1 in response to H_2_O_2_ that suggests a physiological role for this PTM in regulating PDHA1 (Figure 2B). The opposing responses, 44% increase in Ac and 53% decrease in Ub of this target protein suggests that PTM crosstalk could be operating to regulate PDHA1 activity under the conditions described. Interestingly, the percent of PDHA1 that is acetylated (6.1% UT and 3.7% H_2_O_2_) is much higher than the percent of ubiquitinated protein (0.13% UT and 0.19% H_2_O_2_) suggesting that the PTMs are not quantitatively related; however, as there are 12 Ac lysines and only a single Ub lysine reported in the literature the exact quantitative relationship between these PTMs remains to be determined. The last point raises an important issue regarding the interpretation of pan IP enrichment data, if there are multiple copies of a given PTM on a target protein, a situation that is very common, then bona fide changes in PTM levels could be masked or underrepresented under some conditions, therefore identification of a PTM that does not show a change under the experimental conditions used should not automatically rule out a regulatory function. Better site-specific tools; for example, site specific antibodies would be beneficial when examining site-specific modifications physiologically.

Another target protein showing decreased Ac (47%) and corresponding increases in Ub (30.3%) was IDI1 (Figure 4A,B,E). Interestingly, this protein’s PTM profile also indicated a dramatic reduction in phosphotyrosine (65.1%) (Figure 4D,E). The available PTM data in the literature for this target is derived from MS analysis and shows that a single phosphotyrosine, four acetyl lysines and six ubiquitinated lysine residues have been identified in a variety of systems [44,51]. We report the first identification of temporal PTM changes in IDI1 which strongly suggests that IDI1 is regulated by PTMs and possibly involves PTM crosstalk. In this regard 3 of the 4 Ac sites and the 6 Ub sites have been reported to occur on the same lysines (K113, K192 and K223) (phosphor site plus), making competitive crosstalk possible.

The two final target proteins selected for potential crosstalk analysis, ATIC and PDHB, both exhibited a similar PTM profile in that Ac decreases (22.8% and 33.9% respectively) while SUMO 2/3 increases (36.9% and 1425% respectively). As far as we are aware, this is the first report of either ATIC or PDHB being modified by SUMO 2/3 and it is the first report demonstrating opposing temporal changes between Ac and SUMOylation in either of these proteins. Crosstalk between SUMOylation (SUMO 1) and acetylation for the regulation of p53 has previously been reported [52], and a more recent paper describes wide ranging cross talk between SUMOylation and other PTMs, including Ac [53]. Follow-up studies are planned to define the physiologic role of SUMOylation, as well as the SUMO 2/3 and Ac crosstalk for ATIC and PDHB.

Throughout this study, we noted the relatively low stoichiometry of any given PTM. Ac was the most abundant PTM in all mitochondrial proteins in this study which may reflect the relatively large number of Ac modifications on each protein (Table 1). Low stoichiometry (<1–5%) that translates to critical and regulated biological responses is a norm for PTMs and is one of the reasons that it is technically challenging to study these modifications [54,55,56]. The robust Signal-Seeker tools can detect PTMs as low as 11 molecules per cell [26], which is essential as many PTMs are highly dynamic. This may be particularly important for mitochondrial proteins as the stoichiometry of their PTMs appears to be quite low [57,58]. The ability to detect these small, but potentially meaningful changes are critical, as measuring endogenous changes of PTMs are necessary to understand the physiologic relevance of the modification.

PTM mechanisms continue to be identified as critical to the function of a rapidly growing number of proteins and pathways; it is important for non-PTM experts to have access to robust and simple tools that allow them to quickly and efficiently determine if PTMs are playing a role in the regulation of their protein/system of interest. We have validated a set of robust and simple tools that fulfill this need and should allow basic characterization of major PTMs to be a standard operation in any bio lab.

## Figures and Tables

**Table 1:** Mitochondrial Target Proteins.

	Mito-Carta 2.0 ID	Mitochondria Reference	Phospho-Site Plus Ac-K ID:	Number of Ac-K Sites	Target Ac-K Reference	Endogenous Ac-K ID with Signal-Seeker	H_2_O_2_ Induced Decrease in Ac-K	*p*-Value
PDHA1	Yes	36	Yes	12	33	Yes	44.0%	0.016
IDI1	Yes	None	Yes	5	None	Yes	47.0%	0.001
PDHB	Yes	37	Yes	7	None	Yes	33.9%	0.005
MRPL24	Yes	38	Not in humans	1	None	Yes	49.9%	0.013
ATIC	Yes	39	Yes	5	None	Yes	22.8%	0.035
PRDX2	Yes	40	Yes	9	45	Yes	42.4%	0.005
DTYMK	Yes	None	Yes	5	None	Yes	28.9%	0.004
SSBP1	Yes	41	Yes	5	None	Yes	5.1%	0.789
HK2	Yes	42	Not in humans	1	None	Yes	−30.4%	0.667
DLD	Yes	43	Yes	22	None	Yes	−5.0%	0.880

**Table 2:** PTM crosstalk for Mitochondrial Targets.

	Signal-Seeker ID	Ac-K	Ub	SUMO 2/3	pY
PDHA1	Endogenous ID	Yes	Yes	No	No
	Response to H_2_O_2_	↓ 35.8%	↑ 53%	n/a	n/a
IDI1	Endogenous ID	Yes	Yes	No	Yes
	Response to H_2_O_2_	↓ 47.0%	↑ 30.3%	n/a	65.10%
ATIC	Endogenous ID	Yes	No	Yes	Yes
	Response to H_2_O_2_	↓ 22.8%	n/a	↑ 36.9%	No Change
PDHB	Endogenous ID	Yes	Yes	Yes	No
	Response to H_2_O_2_	↓ 33.9%	Trend ↑	↑ 1425%	n/a

## Supplementary Materials

The supplementary materials are available online at http://www.mdpi.com/2227-7382/6/2/24/s1.
