# Supplementary material for: Utilizing Optimized Tools to Investigate PTM Crosstalk: Identifying Potential PTM Crosstalk of Acetylated Mitochondrial Proteins
Source: Proteomes. 2018 May 22;6(2):24. doi: 10.3390/proteomes6020024 (PMC6027404; doi:10.3390/proteomes6020024)
Supplement: Supplementary file 1 [file proteomes-06-00024-s001.zip › Supplementary Figures Proteomes Horita Revision.pdf]

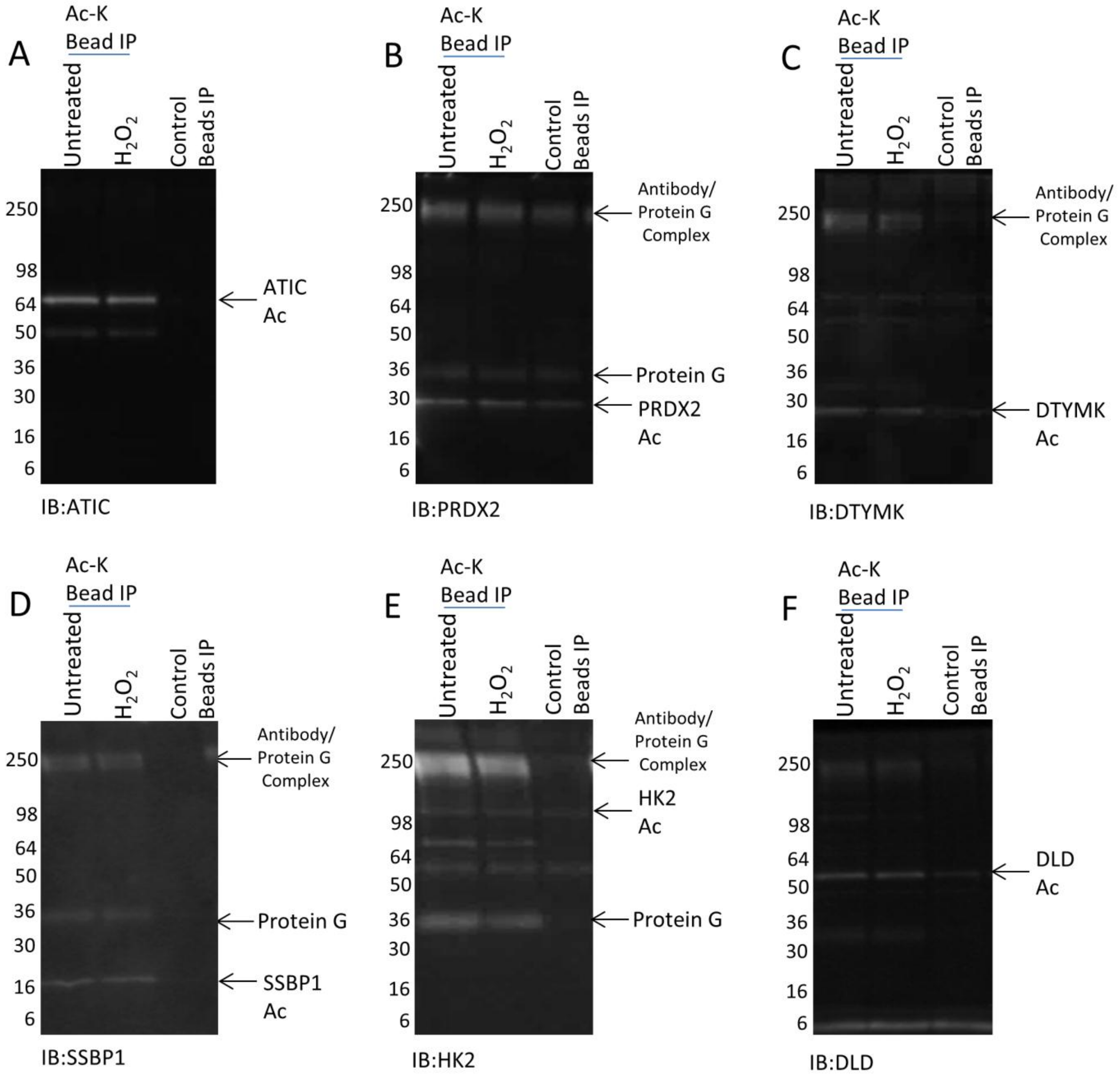

Supplementary Fig 1. Endogenous acetylation of mitochondrial targets. A431 cells either untreated or treated with H_2O_2 for 2 hours were lysed with BlastR lysis buffer. IP of acetylated proteins from 800 μg - 1 mg of lysate were performed using Ac-K Affinity beads or Acetyl-lysine IgG control beads. Eluted proteins were resolved in an SDS-PAGE gel and then transferred to a PVDF membrane. Western blots were performed with (A) ATIC, (B) PRDX2, (C) DTYMK, (D) SSBP1, (E) HK2, and (F) DLD antibodies. Shown are representative westerns from N≥3 independent experiments.

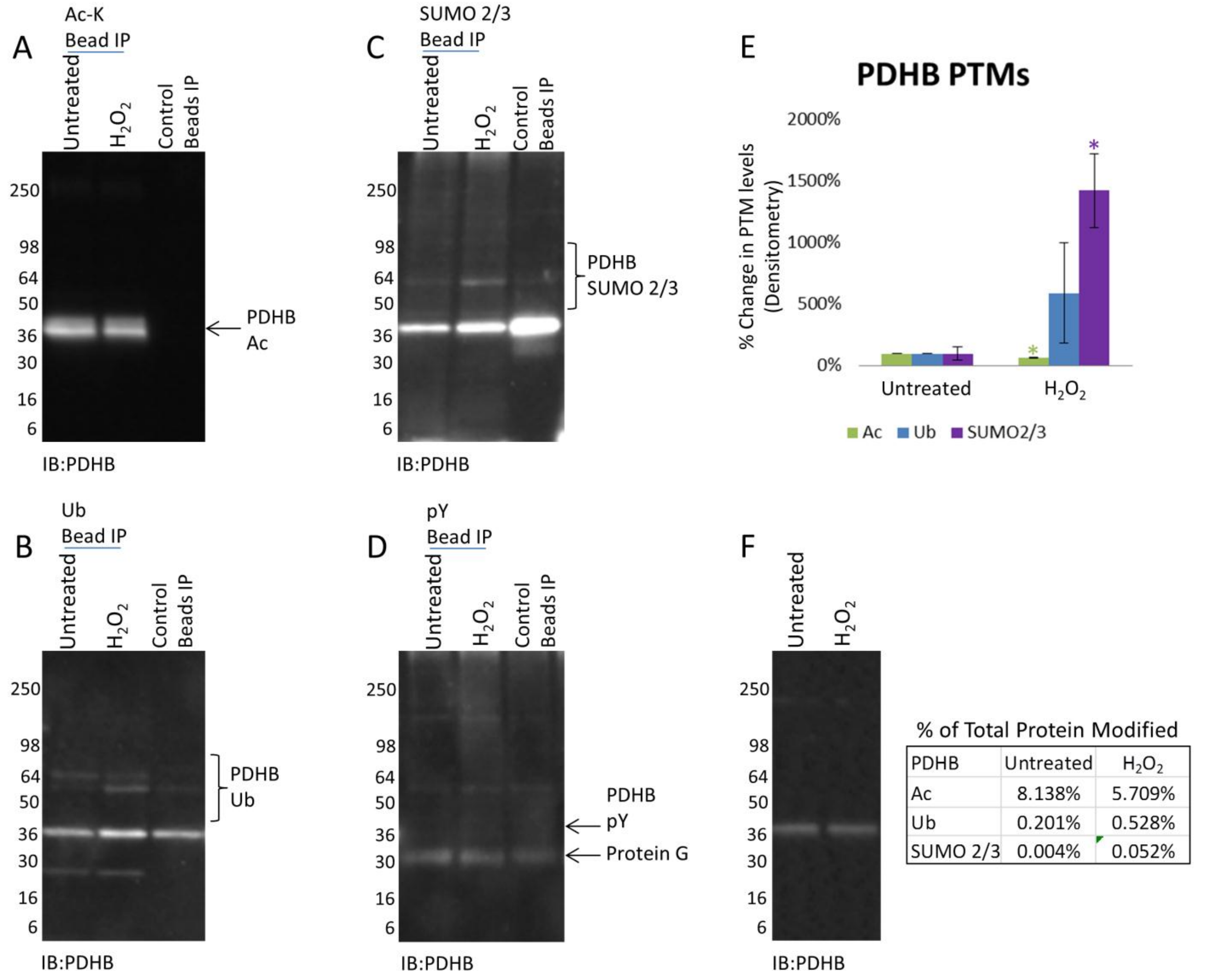

PDHB	Untreated	H_2O_2
Ac	8.138%	5.709%
Ub	0.201%	0.528%
SUMO 2/3	0.004%	0.052%

Supplementary Fig 2. H_2O_2 induced Ac, Ub, SUMO 2/3, and pY modifications of PDHB. A431 cells either untreated or treated with H_2O_2 for 2 hours were lysed with BlastR lysis buffer. Untreated and treated A431 lysates were incubated with (A) Ac-K beads to IP acetylated proteins and analyzed for acetylated PDHB, (B) Ub beads to IP ubiquitinated proteins and analyzed for ubiquitinated PDHB, (C) SUMO 2/3 beads to IP SUMOylated 2/3 proteins and analyzed for SUMO 2/3 modified PDHB, (D) and pY beads to IP tyrosine phosphorylated proteins and analyzed for tyrosine phosphorylated PDHB. All IPs were performed with appropriate control beads to detect non-specific detection. Shown are representative westerns from N≥3 independent experiments. (E) Quantification of background subtracted densitometric analysis of PDHB PTMs. Error bars represent s.e.m. T-test statistical analysis was performed. *$P<0.05$. (F) WCL was analyzed for PDHB levels. The percentage of PTM modified PDHB relative to the total PDHB levels for each modification are shown.

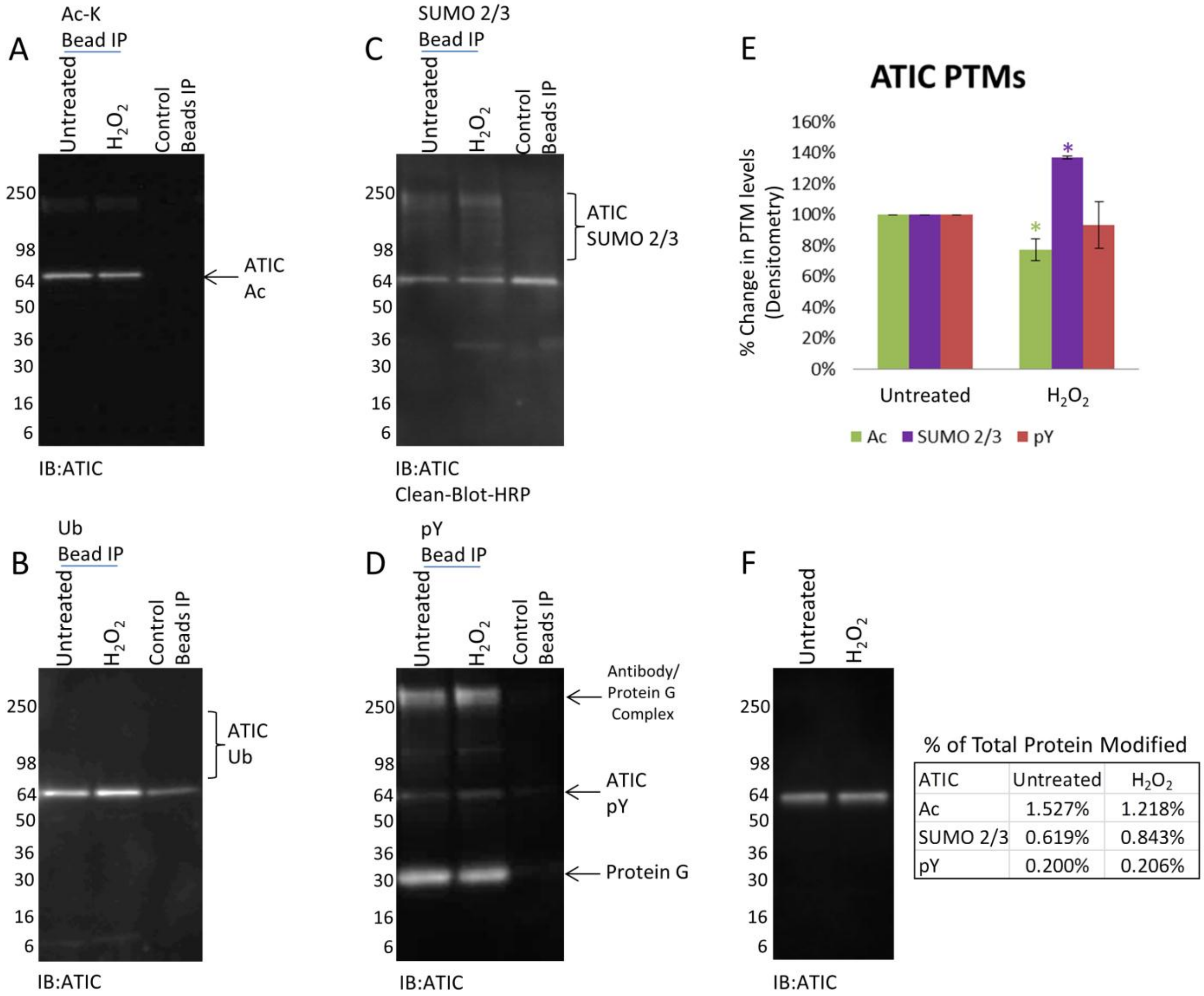

ATIC	Untreated	H_2O_2
Ac	1.527%	1.218%
SUMO 2/3	0.619%	0.843%
pY	0.200%	0.206%

Supplementary Fig 3. H_2O_2 induced Ac, Ub, SUMO 2/3, and pY modifications of ATIC. A431 cells either untreated or treated with H_2O_2 for 2 hours were lysed with BlastR lysis buffer. Untreated and treated A431 lysates were incubated with (A) Ac-K beads to IP acetylated proteins and analyzed for acetylated ATIC, (B) Ub beads to IP ubiquitinated proteins and analyzed for ubiquitinated ATIC, (C) SUMO 2/3 beads to IP SUMOylated 2/3 proteins and analyzed for SUMO 2/3 modified ATIC, (D) and pY beads to IP tyrosine phosphorylated proteins and analyzed for tyrosine phosphorylated ATIC. All IPs were performed with appropriate control beads to detect non-specific detection. Shown are representative westerns from N≥3 independent experiments. (E) Quantification of background subtracted densitometric analysis of ATIC PTMs. Error bars represent s.e.m. T-test statistical analysis was performed. *$P<0.05$. (F) WCL was analyzed for ATIC levels. The percentage of PTM modified ATIC relative to the total ATIC levels for each modification are shown.
